# Biallelic Nonsense Variants in *NEFL* May Cause a Non‐Length‐Dependent Neuropathy With Temporal Dispersion on Nerve Conduction Studies

**DOI:** 10.1111/jns.70119

**Published:** 2026-03-29

**Authors:** Marcus Vinícius Vieira da Silva Gomes, Pedro José Tomaselli, Wilson Marques

**Affiliations:** ^1^ Department of Neurosciences and Behaviour Sciences, Neuromuscular Disorders University of São Paulo Ribeirão Preto São Paulo Brazil; ^2^ INCT Translational Medicine Porto Alegre Brazil

**Keywords:** autosomal‐recessive Charcot–Marie‐tooth, demyelinating neuropathy, *NEFL*, non‐length‐dependent neuropathy, temporal dispersion

## Abstract

**Background and Aims:**

Pathogenic variants in *NEFL* , the gene that encodes the light polypeptide subunit of neurofilaments, are an uncommon cause of autosomal recessive Charcot‐Marie‐Tooth (CMT) disease. In this study, we describe the clinical and electrophysiological features of two families with early‐onset CMT carrying nonsense variants in the *NEFL* gene.

**Methods:**

Clinical, genetic, and electrophysiological data were collected prospectively and systematically analyzed.

**Results:**

Five patients from two unrelated families were included. All patients had combined proximal and distal muscle weakness and facial weakness. In two individuals, marked proximal weakness of the upper limbs with preserved proximal strength in the lower limbs was observed, suggesting a non–length‐dependent pattern of involvement. Pinprick sensation was preserved in all cases, whereas vibration sense was reduced, mainly distally. Nerve conduction studies demonstrated a demyelinating neuropathy with temporal dispersion in all patients. Whole‐exome sequencing of the probands identified two distinct homozygous pathogenic nonsense variants in *NEFL*: c.54G>C; p.Tyr18* in proband I‐1 and c.796G>T; p.Glu266* in proband II‐1. Sanger sequencing confirmed segregation of the respective variants in affected siblings.

**Interpretation:**

This study suggests that recessive nonsense variants in *NEFL* may cause a non‐length‐dependent sensory and motor neuropathy with facial involvement and temporal dispersion on nerve conduction studies, mimicking an acquired inflammatory demyelinating neuropathy.

## 1 Background and Aims

Charcot–Marie‐Tooth (CMT) and related disorders are a group of clinically and genetically heterogeneous inherited peripheral neuropathies characterized by distal muscle weakness and atrophy, sensory impairment, areflexia, and skeletal deformities. These disorders display different patterns of inheritance, including autosomal dominant (AD), autosomal recessive (AR), and X‐linked patterns. To date, more than 130 genes have been implicated in CMT [1]. However, variants in *PMP22*, *GJB1*, *MFN2*, and *MPZ* collectively account for nearly 90% of all diagnosed cases [2]. According to upper‐limb motor nerve conduction velocity (MNCV), three presentations can be identified: a demyelinating group (MNCV < 35 m/s), an axonal group (MNCV > 45 m/s), and an intermediate form (MNCV between 35 and 45 m/s) [3].

*NEFL* is a rare (< 1%) but well‐established causative gene of CMT [4]. The *NEFL* gene encodes the light polypeptide subunit of neurofilaments, one of the three major structural components of the neuronal cytoskeleton, providing mechanical stability to neurons in both the peripheral and central nervous system [5]. Pathogenic variants in *NEFL* result in failure to assemble with wild‐type proteins or to self‐assemble, thereby disrupting the neurofilament network and impairing axonal transport [6]. The most common mutations in *NEFL* are missense variants, typically located in the head domain or at the N‐ and C‐terminal ends of the rod domain [7].

Its clinical presentation is highly variable, ranging from a mild oligosymptomatic sensorimotor neuropathy to a severe early‐onset form with loss of ambulation and central nervous system involvement (CNS) [8]. Most cases follow an AD inheritance pattern, although a few AR cases have been reported [9]. Animal studies and clinical reports consistently link *NEFL* mutations to the axonal/intermediate subtype (CMT2E/CMT‐DIG), although demyelinating cases (CMT1F) have also been described [10].

In this report, we describe two families descended from Indigenous populations of the Amazon rainforest who present with early‐onset autosomal recessive demyelinating CMT caused by two novel nonsense variants in *NEFL*.

## 2 Case Report

Both families had ancestors from Indigenous tribes in the Amazon rainforest of northern Brazil and exhibited admixture with Portuguese migrants.

**FIGURE 1:**
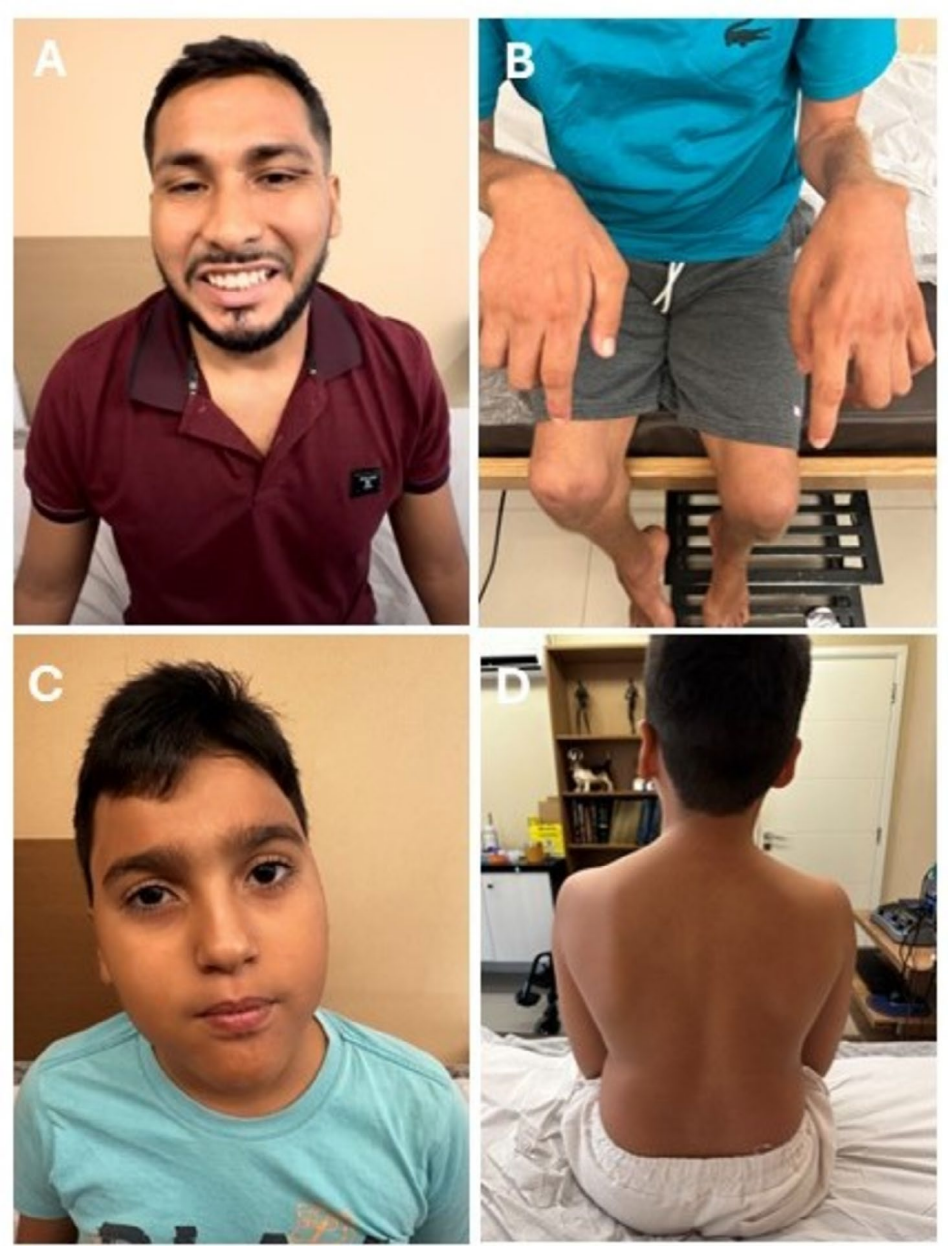
Clinical features. Mild facial weakness in patients I‐1 (A) and II‐1 (C). Severe muscle atrophy of the forearms and intrinsic hand muscles in patient I‐2 (B). Mild asymmetric scapular winging (D) in patient II‐1.

**FIGURE 2:**
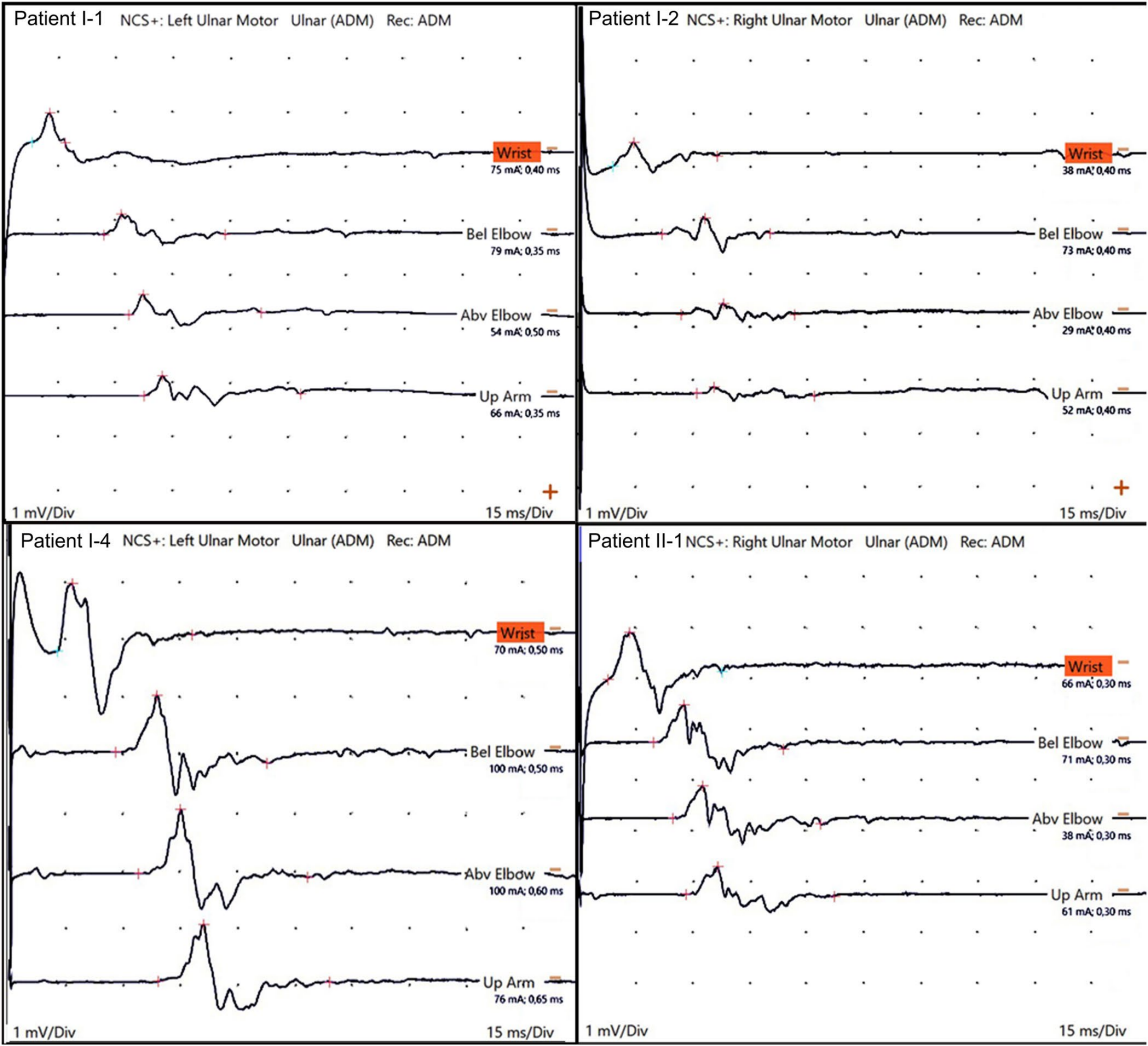
Electrophysiological features. CMAP of the ulnar nerve showing temporal dispersion in patients I‐1, I‐2, I‐4, and II‐1.

The proband of family I (I‐1) had normal neurodevelopmental milestones. At the age of 7 years old, he began with gait difficulties and frequent falls. His parents were clinically normal and denied any known consanguinity, although belonging to the same village. Symptoms progressed over the years, and by the age of 15, he noted a slight inversion of the left foot. Subsequently, he developed a tremor in both hands, bilateral facial weakness, and required unilateral support for ambulation by the age of 27. Neurological examination at 31 years of age revealed symmetrical proximal (MRC grade 3/5) and distal (0/5) muscle weakness in the upper limbs bilaterally, associated with mild facial weakness (Figure 1). In the lower limbs, there was no proximal weakness, but foot dorsiflexion was graded MRC 0/5 bilaterally, and foot plantarflexion was 1/5 on the left and 0/5 on the right (Table 1). Muscle atrophy was observed below the knees and in both the proximal and distal muscles of the upper limbs, including the triceps, biceps, deltoid, and intrinsic hand muscles. Deep tendon reflexes were globally absent. Vibration sense was reduced up to the wrists in the upper limbs and up to the knees in the lower limbs, while pinprick sensation was preserved. A tremor was observed in his voice and hands. He also exhibited pes cavus and equinovarus posture of the left foot. Motor nerve conduction studies (MNCS) demonstrated a severe reduction in amplitude and conduction velocity, temporal dispersion, and bilateral prolonged R1 and R2 on blink reflex testing (Figure 2). The Charcot–Marie‐Tooth neuropathy score (CMTNSv2) was 21. There were two affected brothers (I‐2 and I‐3) and a second‐degree cousin (I‐4) who presented with a similar clinical phenotype, with disease onset in the first decade of life. One of the brothers (I‐2) also demonstrated marked proximal weakness of the upper limbs (MRC 2/5 bilaterally), with relative preservation of proximal strength in the lower limbs, and a pattern of muscle atrophy like that observed in patient I‐1 (Video S1).

**TABLE 1:** Clinical, genetic, and electrophysiological features of patients included in this study.

Patient	I‐1	I‐2	I‐3	I‐4	II‐1
Gene	*NEFL*	*NEFL*	*NEFL*	*NEFL*	*NEFL*
Zigosity	Homozigous	Homozigous	Homozigous	Homozigous	Homozigous
Variant	c.54G>C; p.Tyr18*	c.54G>C; p.Tyr18*	c.54G>C; p.Tyr18*	c.54G>C; p.Tyr18*	c.796G>T; p.Glu266*
Type	Nonsense	Nonsense	Nonsense	Nonsense	Nonsense
Sex	M	M	M	M	M
Age of onset	7 years	8 years	8 years	2 years	1 year
Age at neurological examination	31 years	44 years	38 years	19 years	10 years
Facial weakness	Mild asymmetrical	Mild	Mild	Mild	Mild
Arm abduction (R/L)	3/3	2/2	4/4	4/4	4−/4−
Arm flexion (R/L)	3/3	3/4—	4/4	4+/4+	4−/4−
Wrist extension (R/L)	3/3	0/3	3/3	4−/4−	2/2
FDI and APB (R/L)	0/0	0/0	1/1	1/1	0/0
Hip flexion (R/L)	5/5	5/4—	4+/4+	4/4	4+/4+
Knee extension (R/L)	5/5	5/5	4+/4+	4/4	4+/4
Foot dorsiflexion (R/L)	0/0	0/0	0/0	0/0	0/0
Foot plantarflexion (R/L)	0/1	0/0	0/0	0/0	0/0
Reflexes in UL/LL	A/A	A/A	A/A	A/A	A/A
Vibration in UL/LL	↓, Wrists/↓, Knees	↓, Wrists/↓, Knees	Normal/↓, Ankles	Normal/↓, Knees	↓, Wrists/↓, Knees
Pain or temperature in UL/LL	Normal/Normal	Normal/Normal	Normal/Normal	Normal/Normal	Normal/Normal
Mental development	Normal	Normal	Normal	Normal	Learning difficulties
CMTNSv2	21	24	25	21	26
Ulnar nerve MCV (m/s)	14	15	12	13	17
Ulnar nerve CMAP amplitude (mV)	0.11	0.45	0.28	1.54	0.91
CMAP temporal dispersion	Yes	Yes	Yes	Yes	Yes
Additional features	Hand and facial tremor, pes cavus, left equinovarus foot	Pes cavus	Pes cavus, Hand tremor	Pes cavus, Hand tremor	Pes cavus, Scoliosis, cognitive deficit

Abbreviations: ↓, reduced; A, absent; APB, abductor pollicis brevis; CMAP, compound motor action potential; CMTNSv2, Charcot–Marie‐Tooth neuropathy score version 2; D, diminished; FDI, first dorsal interosseous; L, left; LL, lower limbs; M, male; MCV, motor conduction velocity; *NEFL*, neurofilament light‐chain polypeptide; R, right; UL, upper limbs.

The proband of family II (II‐1) was the only child born from healthy first‐cousin parents. Pregnancy and birth occurred uneventfully, but motor milestones were delayed: he was unable to stand until the age of 2 years old and did not walk independently until 2.5 years of age. Neurological examination at the age of 10 revealed both proximal and distal muscle weakness, bilateral foot drop, pes cavus, facial weakness, and scoliosis (Table 1). He needed ankle orthotics and bilateral support for ambulation since the age of two. Muscle atrophy was observed in the lower limbs from the thighs distally and in the upper limbs from the forearms distally, with additional deltoid wasting, accompanied by asymmetric scapular winging (Figure 1). Although he reported no sensory complaints, vibration sense was decreased up to the knees and wrists. Deep tendon reflexes were absent throughout. There were no pyramidal signs or hearing loss. He exhibited mild cognitive deficits and learning difficulties. The MNCS and blink reflex had the same pattern described in proband I‐1 (Figure 2). The CMTNSv2 was 26.

Whole‐exome sequencing (WES) was performed on both probands and identified two different homozygous nonsense variants in the *NEFL* gene (NM_006158.5), both classified as pathogenic (class 5) according to ACMG criteria. Both variants identified, c.54G>C; p.Tyr18* in proband I‐1 and c.796G>T; p.Glu266* in proband II‐1, are extremely rare in gnomAD and introduce premature stop codons at positions 18 and 266, respectively. Notably, the c.796G>T; p.Glu266* variant is novel, with loss of function as the defined pathogenic mechanism. Written informed consent was obtained from the patients for participation in this study and for the publication of clinical data and identifiable images included in this manuscript. The study was approved by the local Ethics Committee.

## 3 Interpretation

We report the first two cases of autosomal recessive *NEFL*‐related CMT in the Amazon region of Brazil. The population in this region is characterized by low rates of migration, with families remaining in the same geographic area for generations. Additionally, the region's tradition of close‐knit communities has led to a higher rate of consanguineous unions, which are culturally accepted and partly reflect the Indigenous heritage, contributing to a higher frequency of autosomal recessive conditions.

The typical clinical phenotype associated with *NEFL*‐related neuropathy is a length‐dependent sensorimotor neuropathy, with onset generally occurring early, often before three years of age; however, reported onset ages range widely from 1 to 40 years [11]. Early‐onset cases usually present with greater severity, often manifesting as delayed motor milestones, difficulty running or walking, hypotonia, and areflexia, as observed in case II‐1. While mild and oligosymptomatic presentations have been described in some individuals, others may progress to more severe forms, leading to loss of ambulation and multisystem neurological involvement. CNS manifestations may include spasticity, cerebellar ataxia, nystagmus, dysarthria, and, occasionally, cerebellar atrophy on neuroimaging, particularly in association with specific variants [8]. Additional features such as sensorineural hearing loss, urinary dysfunction, and cardiac conduction abnormalities have also been reported [12].

Patients I‐1 and I‐2 presented in the first decade of life with facial weakness and disproportionate proximal weakness in the upper limbs, with normal or nearly normal proximal strength in the lower limbs, suggesting a non–length‐dependent pattern. In our cohort, the distribution of weakness does not conform to the classical length‐dependent pattern. Although distal weakness predominated overall, several clinical features are inconsistent with a strictly distal‐to‐proximal pattern gradient. In proband I‐1, there was moderate (MRC grade 3) proximal upper‐limb and facial weakness in the absence of proximal lower‐limb involvement (MRC grade 5), which is difficult to reconcile with the expected preferential involvement of the longest motor axons, in which intrinsic foot muscles are typically affected first, followed by peroneal‐innervated muscles and only later the gastrocnemius [13].

In the remaining individuals, proximal weakness in the lower and upper limbs was similarly severe. We did not observe greater weakness in the lower limbs compared with the upper limbs, as would be expected in a strictly length‐dependent process. Taken together, early facial involvement, disproportionate proximal upper‐limb weakness, and the absence of a consistent gradient favoring the longest motor axons support a regionally selective, patchy pattern of motor involvement rather than a typical length‐dependent neuropathy.

Another notable clinical feature was the preservation of thermoalgesic sensations, accompanied by marked involvement of vibration, consistent with previous reports [14]. In the patients we had the opportunity to evaluate, the c.796G>T; p.Glu266* variant in patient II‐1 was associated with a more severe neuropathy, but to reach a more definite conclusion, other patients should be available.

*NEFL*‐associated neuropathy has a well‐recognized but heterogeneous electrophysiological profile, characterized by a dominant axonal loss, often accompanied by conduction slowing, frequently in the intermediate range, and prolonged distal latencies [10, 15, 16]. This slowing is usually non‐uniform and may overlap with demyelinating values, but is generally interpreted as secondary myelin dysfunction on a primary axonal background. Consistent with this concept, marked temporal dispersion is not typically observed in *NEFL*‐related disease. In our recordings, we excluded technical artefacts and a superimposed immune‐mediated demyelinating neuropathy.

Neurofilament light is predominantly expressed in neurons and localizes to axons in both the central and peripheral nervous systems. In peripheral nerves, *NEFL* forms part of the neurofilament heteropolymer, a major cytoskeletal component of large myelinated axons, where it contributes to structural support, maintenance of axonal caliber, and efficient impulse conduction by stabilizing the axonal cytoskeleton and internodal organization [17]. Neurofilaments also interact with microtubules and other cytoskeletal elements, influencing axonal mechanical properties and resilience. Although neurofilament expression is usually restricted to neurons, transient *NEFL* mRNA and protein expression has been reported in Schwann cells after peripheral nerve injury or demyelination, particularly when axonal contact is lost, and is thought to support regeneration and repair [18].

Nerve biopsies from patients with *NEFL*‐related CMT typically show a chronic axonal neuropathy with secondary demyelinating changes. Common findings include loss of large myelinated fibers, axonal degeneration with clusters of regenerating axons, and, in a subset of cases, onion‐bulb formations and/or thinly myelinated fibers [17]. Focal accumulations of neurofilaments with axonal swellings have been described and reflect disruption of the axonal cytoskeleton [18]. On electron microscopy, reduced neurofilament density and smaller axonal caliber may be seen, sometimes with an apparent increase in microtubules and absence of neurofilament aggregates in distal segments [19]. Overall, the morphological picture is that of a length‐dependent chronic axonal neuropathy in which disturbance of the neurofilament network leads to axonal degeneration, with variable degrees of secondary demyelination and remyelination, as suggested by the presence of onion bulbs in some, but not all cases. These biopsy features are consistent with the central role of *NEFL* in organizing the axonal cytoskeleton and support the hypothesis that *NEFL* mutations cause neuropathy primarily by disrupting axonal architecture and maintenance. In our patients, however, the clear demonstration of temporal dispersion suggests additional involvement of nodal and internodal structures, indicating a more complex disturbance of axon–myelin interaction than is typically recognized in *NEFL*‐related disease.

Although there was a marked axonal degeneration, the combination of MNCV and blink reflexes suggests a demyelinating neuropathy. Unfortunately, we were unable to perform nerve biopsies.

Our findings suggest that recessive nonsense variants in *NEFL* may cause a non‐length‐dependent sensorimotor neuropathy with facial involvement, preservation of thermoalgesic sensations, and a demyelinating pattern on NCS with temporal dispersion. This description includes *NEFL* among the growing number of genes associated with non‐length‐dependent inherited demyelinating neuropathies with non‐uniform conduction, a potential source of misdiagnosis in acquired inflammatory demyelinating neuropathies.

## Conflicts of Interest

The authors declare no conflicts of interest.

## Supporting information


**Video S1:** jns70119‐sup‐0001‐VideoS1.mp4.

## Data Availability

The data that support the findings of this study are available from the corresponding author upon reasonable request.

## Appendix A

Separate caption for Video S1:

00:03 to 00:05—Patient I‐2.

00:05 to 00:17—Arm abduction: 2/5 (right), 2/5 (left).

00:18 to 00:27—Arm flexion: 3/5 (right), 4−/5 (left).

00:28 to 00:37—Wrist extension: 0/5 (right), 3/5 (left).

00:38 to 00:54—Abductor pollicis brevis and first dorsal interosseous: abduction 0/5 (right), 0/5 (left).

00:54 to 01:04—Hip flexion 5/5 (right), 4−/5 (left).

01:04 to 01:18—Hip adduction: 5/5 (right), 5/5 (left). Knee extension: 5/5 (right), 5/5 (left).

01:19 to 01:30—Ankle dorsal and plantar flexion 0 bilaterally.
